# The effect of wearing face masks on voice and intelligibility of speech during the COVID-19 pandemic

**DOI:** 10.1186/s43163-023-00410-6

**Published:** 2023-03-08

**Authors:** Aisha Fawzy Abdel-Hady, Hossam Mohamad El Dessouky, Hagar Hussein Saqr, Heba Mahmoud Farag

**Affiliations:** Phoniatric Unit, Otolaryngology Department, Kasr Al-Ainy, Faculty of Medicine, Cairo University, Cairo, Egypt

**Keywords:** Face masks, Speech intelligibility, Voice parameters, Voice fatigue

## Abstract

**Background:**

The study aims at evaluating the effect of wearing face masks on voice and intelligibility of speech in Egyptian working individuals during the COVID-19 pandemic to identify if there are any adverse effects of wearing face masks in the working environment.

**Materials and methods:**

A cross-section analytical study was conducted on 153 participants. Personal data and data about the nature of their workplaces were collected. The evaluation included a subjective assessment of voice and intelligibility of speech using a specifically designed questionnaire addressing self-perception of voice fatigue, speech unintelligibility, received auditory feedback and breathing difficulty, and objective voice assessment by Computerized Speech Lab, while objective speech unintelligibility assessment by the Arabic Speech Intelligibility Test.

**Results:**

The study revealed poor workplace acoustics and increased their self-perception of voice fatigue, speech unintelligibility, auditory feedback, and breathing difficulty while wearing masks. Medical professionals showed increased self-perception of speech unintelligibility and the received auditory feedback. No significant difference was found in absolute jitter with and without a face mask. Increasing shimmer and mean fundamental frequency and decreasing noise to harmonic ratio and maximum phonation time were found. The study revealed decreased speech intelligibility especially with the N95 mask.

**Conclusion:**

Wearing face masks negatively affects communication in the workplace, with poor room acoustics. It affects both speech intelligibility and voice subjectively and objectively. It caused increased self-perception of voice fatigue and changes in objective voice parameters.

## Background

In 2020, the World Health Organization declared a pandemic caused by the severe acute respiratory syndrome coronavirus 2 (SARS-COV 2) which causes coronavirus disease (COVID-19) in humans. To control disease transmission, several interventions have been recommended at the individual, environmental, and community levels. These interventions include social distancing, proper ventilation of rooms, cleaning surfaces and objects, washing hands regularly, and using personal protective equipment, such as face masks [1, 2].

Face masks are non-pharmacological public health interventions which play a vital role in controlling disease spread. There are various kinds of masks available—medical masks, respirators like N95, and non-medical masks like cloth masks [3]. The most recommended face masks are the surgical mask for professional use or the N95 while performing potentially aerosol-generating procedures, and the three-layer cloth mask for professional activities in order to protect from the contagion and the proliferation of the virus [1].

Unfortunately, face masks can have an adverse effect on speech communication at both ends as listeners may experience a decrease in the speech intelligibility and speakers may experience an increase in vocal effort. The negative effects of wearing a face mask on speech intelligibility (SI) could be even worse in poor acoustic conditions, such as in the presence of high reverberation time and background noise. A secondary effect of wearing face masks is that they interfere with speech-reading and visual speech cues from facial expressions, which is more detrimental to people with hearing impairment [4].

It is known that wearing a face mask causes voice attenuation, which can further lead to an increase in the loudness or vocal intensity. It can also influence other levels of vocal production and generate pneumo-phono-articulatory incoordination. Vocal misuse and abuse resulting from inadequate vocal adjustments and excessive muscle tension may increase the perception of vocal fatigue symptoms, discomfort, and even trigger dysphonia [1].

The adverse effects of wearing face masks are claimed to relate to the amount of voice use. There is a special group of individuals that present high vocal demand; the voice professionals, including singers, teachers, telemarketing operators, lawyers, consultants, salesmen, and healthcare providers [5]. The use of the voice associated with the face masks could produce more symptoms of tiredness and restriction related to the vocal use during professional activities [1]. Potential working environment risk factors for developing voice disorders have been identified for many vocally demanding professions such as extensive use of voice with not enough voice rest time, high background noise, poor workplace acoustics, poor indoor air quality, poor speaking postures, and lack of appropriate technical aids such as sound amplifiers [6].

To our knowledge, there is no study that has been done in Egypt to investigate the effect of wearing face masks on various aspects of communication. This raised the utmost interest to undertake such study in the Egyptian environment to identify if voice and intelligibility of speech as important communication parameters are affected by wearing the face masks especially in working people with different vocal demands and various adverse factors in their workplace.

The study aims at evaluating the effect of wearing face masks on voice and intelligibility of speech in Egyptian working individuals during the COVID-19 pandemic to identify if there are any adverse effects of wearing face masks in the working environment that might hinder effective communication with secondary intention to study the effects on communication according to the type of the used face mask.

## Methods

This study is a cross-section analytical study that was conducted in the Phoniatric Unit, Faculty of Medicine, Cairo University (Kasr Al-Ainy Hospital). The study was ethically approved by the Research Ethical Committee of Cairo University.-Code: MS-148–2021. Written consent was taken from all the participants. The study was conducted in the period between April 2021 and February 2022.

### Population of the study

The study included 153 working Egyptian individuals in occupations that involve direct personal communication. They were recruited from Kasr Al-Ainy visitors such as relatives of patients and workers in the hospital such as doctors, nurses, and employees who met the inclusion criteria of the study. The sample size was calculated using “statistics and sample size pro” based on our primary objective; considering the following data: the mean score of speech intelligibility is 1.97, SD 1.09; the mean score of auditory feedback is 2.68, SD 1.26, with alpha error 0.05; and the power of the study is 80%.

### Inclusion criteria

The participants were selected with the following inclusion criteria: age range between 20 and 55 years, they should be literate (can read and write) with an educational level reaching at least secondary school or diploma, their occupations involve direct personal communication, with no presence of mental disability, no voice disorder prior to the use of face mask, no speech, neurological, or psychiatric disorders affecting communication and no hearing or marked visual problem.

### Methodology in details

The selected participants were interviewed and informed about the idea of the study, and a written consent was taken. They were subjected to history taking to collect the personal data: age, educational level, profession, type of the face mask they tend to use in addition to the adaptation of the face mask on their face if it is comfortable, loose or tight, their daily voice demand by measuring the voice workload in hours/day and the weekly voice workload in days/week, in addition to the level of speech usage during work (the questions were designed in light of the Levels of Speech Usage Categorical Rating Scale) [7]: undemanding (the subject remains quiet for long periods of time almost every day), intermittent (the subject remains quiet for long periods of time on many days—most talking is typical conversational speech), routine (the subject has frequent periods of talking on most days—most talking is typical conversational speech), extensive (the subject’s speech usage consistently goes beyond everyday conversational speech), and extraordinary (the subject has very high speech demands).

Data were collected about the condition of their work place including the health condition of people he/she deals with (if they are normal or with any disorder affecting communication), workplace noise, presence of acoustic treatment to reduce noise (sound absorption on walls, partitions between desks, carpets, or other), and if there is any modification for the workplace as precautions used during the pandemic (Appendix 1).

The participants filled in a questionnaire that was specifically designed in the study in the Arabic language, to evaluate their subjective impression about the effect of wearing the face mask on the following aspects:

1. Self-perception of voice fatigue without a face mask and with face mask (Appendix 2, section I).
  To verify the perception of vocal fatigue without a face mask and with a face mask, the questionnaire included questions (guided by the Vocal Fatigue Index) [8] about 3 main factors:
  1. Tiredness of voice and voice avoidance (including 4 questions).
  2. Physical discomfort associated with voicing (including 2 questions).
  3. Improvement of symptoms with rest (including 1 question).
2. Self-perception of speech unintelligibility with face mask (Appendix 2, section II).
  To analyze the perception of speech unintelligibility, the questionnaire included questions about the subject’s perception of difficult or restricted movements of articulators and the use of compensatory mechanisms such as; raising voice, clarifying speech, repeating utterances, or slowing speech rate, as a response to subject’s self-perception of the presence of a sort of unintelligibility of his/her speech while wearing the face mask.
3. The received auditory feedback from their listeners while the subject is wearing a face mask (Appendix 2, section III).
  To analyze the received auditory feedback from the listeners, the questionnaire included questions about the listeners’ comments on the subject’s speech intelligibility and the subject’s use of compensatory strategies such as; raising voice, clarifying speech, repeating utterances, or slowing speech rate, as the listeners tend to ask the subject for that.
4. Self-perception of breathing difficulty with face mask (Appendix 2, section IV).

To analyze the self-perception of breathing difficulty with a face mask, the questionnaire included questions about: the subject’s self-perception of inspiration difficulty and/or self-perception of expiration difficulty.

The subjects evaluated their self-perception of the previous aspects of self-perception of voice fatigue, speech unintelligibility, breathing difficulty with a face mask on a 4-point scale between zero (never) in case of no difficulty and three in case they always face a difficulty regarding each aspect.

Total scores were calculated for each section in the questionnaire by summation of the scores of questions included in each section.

Before the application of the questionnaire, a pilot study was carried out on a number of 10 subjects within the inclusion criteria of the study to check the comprehensibility of the introduced questions and to estimate the time of the application and for the purpose of the face and content validity. All the questions were found to be easily understood and the average duration was about 15 min.

Each participant underwent an objective assessment of voice through acoustic voice analysis which was performed twice; once with the face mask and once without the face mask.

All voice samples were recorded in a quiet sound-treated room. Each subject was seated in front of a microphone placed 15 cm from his/her mouths. Each subject was asked to produce the sustained vowel /a/ at a comfortable pitch, constant amplitude, and flat tone.

The professional voice recording software of the “Computerized Speech Lab (CSL) Model 4500” (Kay Elemetrics Corporation, Lincoln Park, NJ, USA) was used. Further editing of the recordings was done using the Multi-Dimensional Voice Program (MDVP).

The acoustic analysis was done using the voice analysis software Multi-Dimensional Voice Program (MDVP). The Multi-Dimensional Voice Program (MDVP) is the most used and cited acoustic analysis software [9].

The acoustic analysis software was used to determine: Mean fundamental frequency (MFF), Jitter, Shimmer in dB, noise-to-harmonic ratio with the aerodynamic measurements of the maximum phonation time (MPT).

Speech intelligibility was assessed in all subjects twice; once with the face mask and once without the face mask, using the Arabic Speech Intelligibility Test for Adolescents and Adults (ASIT-AA) [10].

The test is composed of 250 cards carrying 125 words (each word is repeated twice). The words are organized in the form of five sets. Each set consists of 25 phonetically confusable words, all are real words.

The words may differ only in one consonant or one vowel sound. This adds a challenge to the listener to try to discriminate what is being uttered by the subject correctly. The words are divided into five sets that start with various speech sound placements including bilabials and labiodentals, dental and linguoalveolar, alveolopalatal, linguovelar and lingulo-uvular, and pharyngeal and glottal consonants.

The test was applied in a quiet room where the examiner was seated facing the subject. The cards were placed on a table between the subject and the examiner and faced down. Before starting, the cards were scrambled. Then, the subject was asked to pick each word and read it. The examiner did not see the word or look at the subject being tested. Each set of words was tried separately. The examiner wrote down—in order—what he thought was uttered by the subject in a scoring sheet and the cards were placed—in the same order—in a separate box. The cards were matched with the examiner’s sheet, and the number of correct responses was estimated. The number of correct responses in relation to the total number of words (250) was expressed in percentage.

A group of 10 participants was asked to fill in the questionnaire again after 1 month for the purpose of evaluating the test–retest reliability.

### Data management and statistical analysis

All collected data were revised for completeness and accuracy. Precoded data were entered on the computer using the statistical package of the social science software program, version 26 (SPSS) to be statistically analyzed. Data was summarized using the mean and SD for quantitative variables and the number and percent for the qualitative variables. A comparison between qualitative variables was done using chi-square test, while independent *T* test for the quantitative variable which was normally distributed and non-parametric Mann–Whitney tests for the quantitative variables which were not normally distributed. One-way ANOVA was used to compare quantitative variables between more than two categories for the quantitative variable which will be normally distributed, and nonparametric Kruskal–Wallis tests for quantitative variables which were not normally distributed. A *P* value < 0.05 was considered significant.

## Results

### Demographic data

The age range of participants was between 20 and 55 years old with the mean age being 34 years. Males constitute 36.6% while females constitute 63.4%. The educational level is “diploma” in 37.9% of the study subjects, “university education” in 18.3%, and “postgraduate studies” in 43.8% of the study subjects. Individuals working in medical professions constitute 64.7% of the study subjects including (50) 32.67% doctors and (49) 32.02% nurses while individuals working in non-medical professions constitute 35.3% of the study subjects including (15) 9.80% employees, (11) 7.18% secretaries, (10) 6.53% teachers, (5) 3.26% drivers, (4) 2.61% engineers, (4) 2.61% sellers, (4) 2.61% policemen, and (1) 0.65% trainer as shown in Table 1.

**Table 1:** Number and percentages of the participants’ professions

Profession	Number	Percentage
Doctor	50	32.67%
Nurse	49	32.02%
Employees	15	9.80%
Secretary	11	7.18%
Teacher	10	6.53%
Driver	5	3.26%
Engineer	4	2.61%
Seller	4	2.61%
Policeman	4	2.61%
Trainer	1	0.65

### Workload distribution

The minimum daily workload among the study subjects is 4 h/day, while the maximum daily workload among the study subjects is 24 h/day, and the mean daily workload is about 8 h/day. The minimum weekly workload among the study subjects is 2 days/week, while the maximum weekly workload among the study subjects is 7 days/week, and the mean weekly workload is 5.35 days.

### Frequency of the type of the used face mask and its adaptation

**Fig. 1:**
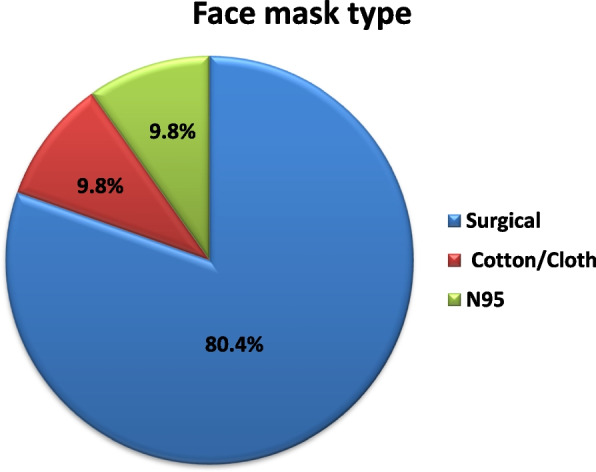
Frequency of the type of face mask used by the study subjects

**Fig. 2:**
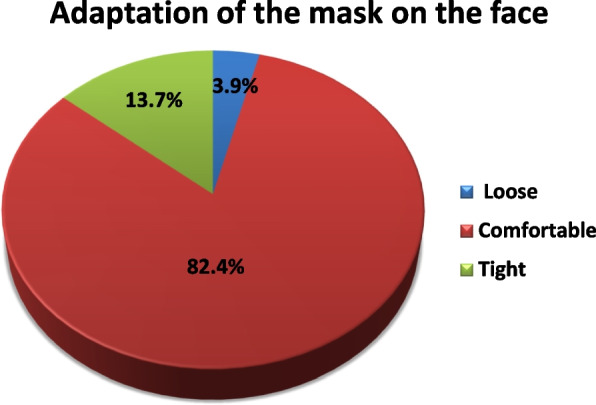
Frequency of mask adaptation on the face among the study subjects

About 80.4% of the study subjects use surgical masks, 9.8% of the study subjects use cloth masks and 9.8% of the study subjects use N95 masks as shown in Fig. 1. The study subjects described the mask adaptation (fit) as being “loose” in 3.9% of them, “comfortable” by 82.4% and “tight” by 13.7% of them as shown in Fig. 2.

### Voice demand and level of speech usage during work

The percentage of voice use per working hours is about 0–25% in 2.6% of the study subjects, 25–50% in 19%, 50–75% in 39.8%, and 75–100% in 38.6% of them as shown in Table 2. The speech usage is described by the study subjects as being “intermittent” by 6.6%, “routine” by 58.8%, and “extensive” by 34.6% of them as shown in Table 3.

**Table 2:** Voice demand during work among the study subjects

		Frequency	Percent
Voice demand during work (percentage of voice use per working hours)	0–25%	4	2.6
	25–50%	29	19
	50–75%	61	39.8
	75–100%	59	38.6

**Table 3:** The levels of speech usage during work among the study subjects

		Frequency	Percent
Speech usage during work	Undemanding	0	0
	Intermittent	10	6.6
	Routine	90	58.8
	Extensive	53	34.6
	Extraordinary	0	0

### The main health condition of people the subjects deal with

**Fig. 3:**
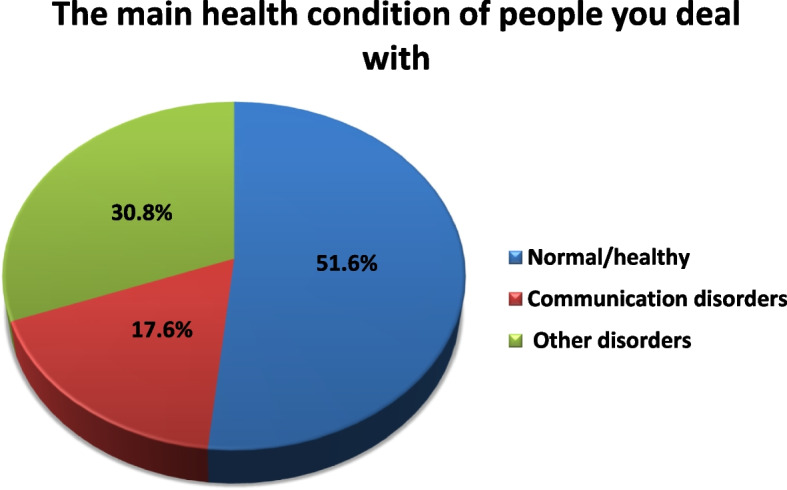
The main health condition of people the subjects deal with

About 51.6% of the study subjects deal mainly with normal individuals, 17.6% of them deal mainly with individuals suffering from communication disorders and 30.8% of them deal mainly with individuals suffering from other medical disorders as shown in Fig. 3.

### Frequency of exposure to noise in the workplace

About 2% of the study subjects “Never” suffer from the presence of noise during work, 46.4% “Sometimes” suffer from the presence of noise during work, 27.4% “Usually” suffer from the presence of noise during work, and 24.2% “Always” suffer from the presence of noise during work as shown in Table 4.

**Table 4:** Frequency of exposure to noise in the workplace

		Frequency	Percent
Workplace noise	Never	3	2
	Sometimes	71	46.4
	Usually	42	27.4
	Always	37	24.2

### Acoustic treatment methods and workplace modifications during the pandemic

**Fig. 4:**
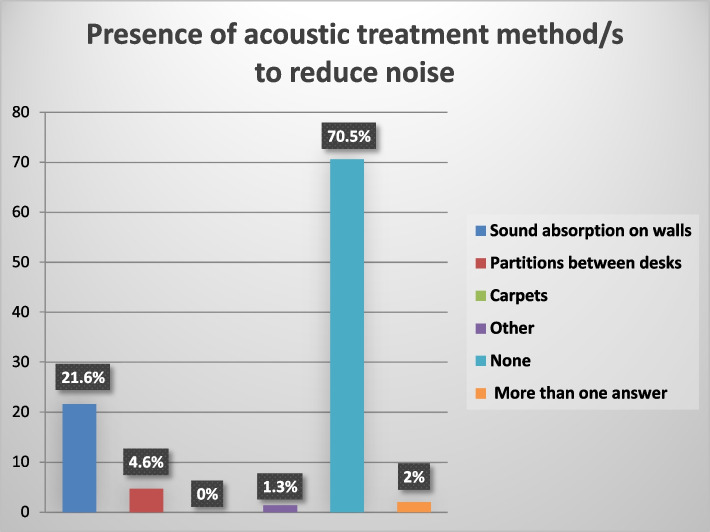
The presence of acoustic treatment methods to reduce noise

**Fig. 5:**
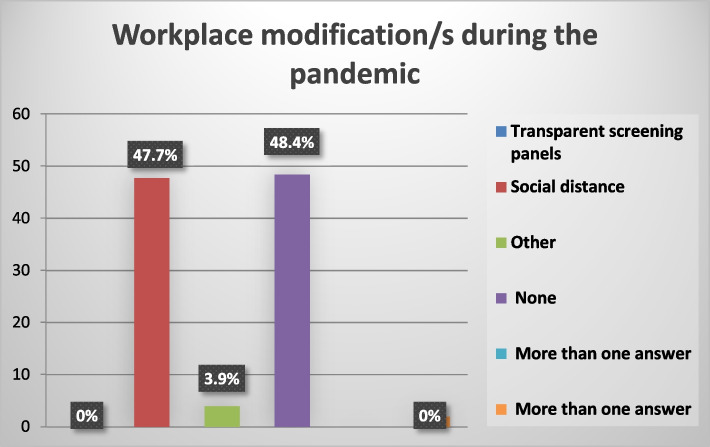
The workplace modifications during the pandemic

“Sound absorption” constitutes 21.6% of the acoustic treatment methods. “Partitions between desks” constitute 4.6%. “Other” like soundproof curtains and acoustic foam constitute 1.3% of the acoustic treatment methods. “None” constitutes 70.5% of the acoustic treatment methods. “More than one answer” constitutes 2% of the acoustic treatment methods as shown in Fig. 4. Social distance” constitutes 47.7% of the workplace modifications.”Other” such as using personal protective equipment as a precaution rather than a workplace modification and decreasing the number of persons whom the study subjects deal with at a time as in limiting the number of caregivers with patients during examination constitutes 3.9% of the workplace modifications. “None” constitutes 48.4% of the workplace modifications as shown in Fig. 5.

### Comparative data

#### Regarding the questionnaire of self-perception of voice fatigue and its sub-items

There is a significant difference between the scores of the questionnaire on self-perception of voice fatigue with and without a face mask regarding the total score of tiredness of voice (*p* value =  < 0.001), the total score of physical discomfort (*p* value =  < 0.001), the score of improvement of voice after rest (*p* value = 0.005), and the total score of self-perception of voice fatigue (*p* value =  < 0.001) with higher scores “with using the face mask” than “without using the face mask” as shown in Table 5.

**Table 5:** Comparison between the total scores of the questionnaire on self-perception of voice fatigue and its sub-items with face mask and without face mask

	With face mask		Without face mask		*P* value
	Mean	SD	Mean	SD	
Total score of tiredness of voice	5.03	2.45	3.09	2.02	< 0.001*
Total score of physical discomfort	1.81	1.44	1.20	1.29	< 0.001*
Score of improvement of voice after rest	0.28	0.59	0.17	0.48	0.005*
Total score of self-perception of voice fatigue	7.12	3.48	4.46	3.01	< 0.001*

^*^Significant *p* value < 0.05, *SD* standard deviation

#### Regarding the measures of voice parameters

There is no significant difference between the measure of absolute jitter “with face mask” and “without face mask” (*p* value = 0.102). There is a significant difference between each of the measures of shimmer (*p* value = 0.001) and the measure of mean fundamental frequency (MFF) (*p* value = 0.013) “with face mask” and “without face mask” with higher values “with using the face mask” than “without using the face mask.” There is a significant difference between each of the measures of noise to a harmonic ratio (N/H ratio) (*p* value =  < 0.001) and the measure of maximum phonation time (MPT) (*p* value =  < 0.001) “with face mask” and “without face mask” with higher values in “without using the face mask” than “with using the face mask” as shown in Table 6. The only significant difference in males is found in the N/H ratio (*p* value = 0.001) with a higher value with a face mask than without a face mask as shown in Table 7. The significant differences are found in females in Shimmer (*p* value = 0.002) with a higher value “with face mask” and in MPT (*p* value =  < 0.001) with a higher value “ without face mask” than “ with face mask” as shown in Table 8.

**Table 6:** Comparison between the measures of voice parameters with face mask and without face mask

	With face mask		Without face mask		*P* value
	Mean	SD	Mean	SD	
Absolute jitter	51.72	33.83	49.03	41.53	0.102
Shimmer in dB	0.27	0.16	0.26	0.21	0.001*
Noise to harmonic ratio (N/H ratio)	0.13	0.02	0.13	0.03	< 0.001*
Mean fundamental frequency (MFF)	179.36	53.73	177.64	53.39	0.013*
Maximum phonation time (MPT)	14.16	5.11	14.85	4.96	< 0.001*

^*^Significant *p* value < 0.05, *SD* standard deviation, *dB* decibel

**Table 7:** Comparison between the measures of voice parameters with a face mask and without a face mask in male participants

Male	With face mask		Without face mask		*P* value
	Mean	SD	Mean	SD	
Absolute jitter	64.91	29.61	63.84	38.52	0.870
Shimmer in dB	0.32	0.20	0.29	0.16	0.090
Noise to harmonic ratio (N/H ratio)	0.14	0.02	0.13	0.03	0.001*
Mean fundamental frequency (MFF)	118.13	18.32	116.81	19.25	0.134
Maximum phonation time (MPT)	17.75	6.03	18.18	5.79	0.060

^*^Significant *p* value < 0.05, *SD* standard deviation, *dB* decibel

**Table 8:** Comparison between the measures of voice parameters with a face mask and without a face mask in female participants

Female	With face mask		Without face mask		*P* value
	Mean	SD	Mean	SD	
Absolute jitter	44.11	33.91	40.49	40.98	0.059
Shimmer in dB	0.25	0.12	0.24	0.23	0.002*
Noise to harmonic ratio (N/H ratio)	0.12	0.02	0.12	0.03	0.058
Mean fundamental frequency (MFF)	214.71	30.49	212.75	29.95	0.056
Maximum phonation time (MPT)	12.08	2.95	12.93	3.09	< 0.001*

^*^Significant *p* value < 0.05, *SD* standard deviation, *dB* decibel

### Regarding ASIT-AA with and without a face mask

There is a significant difference between the scores of all sets; set A (*p* value = 0.001), set B (*p* value =  < 0.001), set C (*p* value =  < 0.001), set D (*p* value =  < 0.001), and set E (*p* value =  < 0.001) of the ASIT-AA “with face mask” and “without face mask” with higher scores “without face mask” than “with face mask.” There is also a significant difference between the total score and percentage of ASIT-AA “with face mask” and “without face mask” (*p* value =  < 0.001) with higher scores “without the face mask” than “with using the face mask” as shown in Table 9.

**Table 9:** Comparison between the ASIT-AA score of each set and the total score with face mask and without face mask

	ASIT-AA score with a face mask		ASIT-AA score without a face mask		*P* value
	Mean	SD	Mean	SD	
Score of set A (bilabial and labiodentals)	49.82	0.52	49.93	0.30	0.001*
Score of set B (interdental, linguo-dental and linguoalveolar consonants)	49.48	0.93	49.84	0.51	< 0.001*
Score of set C (post-alveolar and palatal consonants)	49.44	0.89	49.75	0.59	< 0.001*
Score of set D (linguo-velar and linguo-uvular consonants)	49.71	0.54	49.88	0.34	< 0.001*
Score of set E (pharyngeal and glottal consonants)	49.23	1.10	49.80	0.57	< 0.001*
Total score	247. 66	2.34	249.22	1.19	< 0.001*
Percentage of the total score	99.06	0.93	99.69	0.47	< 0.001*

^*^Significant *p* value < 0.05, *SD* standard deviation, *ASIT-AA* Arabic Speech Intelligibility Test for Adolescents and Adults

### Regarding different sections of the questionnaire and ASIT-AA in different face mask types

There is no significant difference among the three face mask types regarding the total score of self-perception of voice fatigue (*p* value = 0.133), the total score of self-perception of speech unintelligibility with face mask (*p* value = 0.055), and the total score of the received auditory feedback with face mask (*p* value = 0.086). There is a significant difference among the three face mask types regarding the total score of the ASIT-AA (*p* value = 0.046) as shown in Table 10.

**Table 10:** Comparison between face mask types regarding the total scores of self-perception of voice fatigue, self-perception of speech unintelligibility, and the received auditory feedback

	Surgical mask		Cloth mask		N95 mask		*P* value
	Mean	SD	Mean	SD	Mean	SD	
Total score of self-perception of voice fatigue	7.20	3.53	5.53	3.74	8.00	2.17	0.133
Total score of self-perception of speech unintelligibility	7.50	3.24	6.93	3.41	9.80	3.90	0.055
Total score of the received auditory feedback	5.24	3.15	4.60	2.32	7.07	3.22	0.086
ASIT-AA total score with face mask	247.86	2.11	248.07	1.79	245.60	3.52	0.046*

^*^Significant *p* value < 0.05, *SD* standard deviation, *ASIT-AA* Arabic Speech Intelligibility Test for Adolescents and Adults

Post hoc pairwise comparisons between face mask types regarding the total score of ASIT-AA illustrate there is a significant difference in the total score of the ASIT-AA between the following: N95 mask and surgical mask (*p* value = 0.015) with higher scores of speech intelligibility in the surgical mask; N95 mask and cloth mask (*p* value = 0.049) with higher scores of speech intelligibility in the cloth mask, while there is no significant difference between a surgical mask and cloth mask (*p* value = 0.850).

### Regarding measures of voice parameters with different face masks

There is a significant difference among the three face mask types regarding the mean fundamental frequency (MFF) (*p* value = 0.005). There is no significant difference among the three face mask types regarding absolute jitter (*p* value = 0.146), shimmer (*p* value = 0.103), noise to a harmonic ratio (N/H ratio) (*p* value = 0.169), and the maximum phonation time (MPT) (*p* value = 0.721) as shown in Table 11.

**Table 11:** Comparison between face mask types regarding the measures of voice parameters

	Surgical mask		Cloth mask		N95 mask		*P* value
	Mean	SD	Mean	SD	Mean	SD	
Absolute jitter	54.13	35.88	47.72	27.05	35.92	11.91	0.146
Shimmer in dB	0.28	0.17	0.26	0.09	0.20	0.05	0.103
Noise to harmonic ratio	0.13	0.02	0.14	0.03	0.13	0.01	0.169
Mean fundamental frequency	178.79	55.16	153.38	46.41	209.94	30.79	0.005*
Maximum phonation time	14.21	5.20	13.80	5.65	14.07	4.01	0.721

^*^Significant *p* value < 0.05, *SD* standard deviation, *dB* decibel

Post hoc pairwise comparisons between face mask types regarding the mean fundamental frequency (MFF) illustrate that there is a significant difference in the mean fundamental frequency (MFF) between the following: N95 mask and surgical mask (*p* value = 0.035), with higher values in the N95 mask; N95 mask and cloth mask (*p* value = 0.003), with higher values in the N95 mask; while there is no significant difference between a surgical mask and cloth mask (*p* value = 0.210).

Further analysis illustrates the significant difference among the three face masks in male participants in MFF (*p* value = 0.028) with a higher significant value with the use of N95 than surgical mask (*p* value = 0.008) and a higher significant value with the use of N95 than cloth mask (*p* value = 0.018). There are no significant differences among the three face masks in females regarding all the measures of voice parameters.

### Regarding the different sections of the questionnaire and ASIT-AA in medical and non-medical professions

There is no significant difference between medical and non-medical professions regarding the total score of voice fatigue without a face mask (*p* value = 0.963), the total score of self-perception of voice fatigue with a face mask (*p* value = 0.631), the total score of ASIT-AA with a face mask (*p* value = 0.3), and without a face mask (*p* value = 0.8). There is a significant difference between medical and non-medical professions regarding the score of self-perception of speech unintelligibility with a face mask (*p* value = 0.005), and the score of the received auditory feedback with a face mask (*p* value = 0.026) with higher scores in the medical professions as shown in Table 12.

**Table 12:** Comparison between medical and non-medical professions regarding total scores of self-perception of voice fatigue, self-perception of speech unintelligibility with face mask, the received auditory feedback with face mask, and ASIT-AA

	Medical professions		Non-medical professions		*P* value
	Mean	SD	Mean	SD	
Total score of self-perception of voice fatigue without face mask	4.38	2.78	4.59	3.40	0.963
Total score of self-perception of voice fatigue with face mask	7.15	3.09	7.06	4.12	0.631
Total score of self-perception of speech unintelligibility with face mask	8.25	3.05	6.61	3.71	0.005*
Total score of the received auditory feedback with face mask	5.76	2.98	4.63	3.27	0.026*
Total score of ASIT-AA with face mask	247.8	2.14	247.41	2.66	0.3
Total score of ASIT-AA without face mask	249.23	1.14	249.19	1.27	0.8

^*^Significant *p* value, *SD* standard deviation, *ASIT-AA* Arabic Speech Intelligibility Test for Adolescents and Adults

### Test–retest reliability

Cronbach’s alpha testing indicates the questionnaire’s content consistency according to Cronbch’s alpha score. Pearson’s correlation test indicates the questionnaire’s reliability after test–retest. *P* value < 2.2e − 16, 95% CI (0.9900223–0.9925454), and the correlation coefficient is 0.9913752. The correlation coefficient is very near to 1 and *p* value < 0.05 as shown in Table 13.

**Table 13:** Correlation test for test–retest reliability

	No. of items	Results of the questionnaire done for the 1st time	Results for the 2nd time	Level of consistency
Voice fatigue without face mask	10	0.846	0.859	High
Voice fatigue with face mask	10	0.68	0.667	acceptable
Self -Perception of speech intelligibility	7	0.412	0.397	low
The received auditory feedback: With face mask	6	0.691	0.686	acceptable
Breathing with face mask	2	0.838	0.892	High

## Discussion

The study aimed at evaluating the effect of wearing the three most commonly used types of face masks (N95 mask, surgical mask, and cloth mask) on voice and intelligibility of speech in Egyptian working individuals during the COVID-19 pandemic from the perspective of the speakers and the feedback they get from their listeners.

The study also attempted to cover the subjective as well as the objective measures to evaluate from both the perspective of the participants and the clinical perspective. In addition, the study tried to shed light on the working environment of the participants and the voice demands during work, to understand the factors that might affect negatively the effective communication in their work environment especially during the COVID-19 pandemic.

The subjects’ age range was selected to be in the working age range from 20 to 55 years with the mean age being about 34 years. The majority of the study subjects worked in medical professions while (35.3%) of the study subjects worked in non-medical professions such as sellers, teachers, engineers, and employees. Since the study subjects were recruited from Kasr Al-Ainy visitors such as relatives of patients and workers in the hospital such as doctors, nurses, and employees, it was expected that individuals working in medical professions would present a major part of the subjects under the study.

The most commonly used type of face masks in the current study is the surgical mask while the remaining study subjects are divided equally in using cloth masks and N95 masks as shown in Fig. 1. Surgical masks and N95 masks are to protect the user from airborne particles. N95 masks are recommended for health workers conducting aerosol-generating procedures during clinical care of COVID-19 patients [11]. Cloth masks come into place to reduce the demand for N95 or surgical masks [12].

The present study showed that the surgical mask is the most commonly used type by the subjects under the study; as surgical masks are cheaper, more easily available, and can be used by healthcare workers who constitute the major part of the study.

The major part of the study subjects described the adaptation (fit) of the mask on their face as “comfortable,” while the remaining part described it as “tight” and to a lesser extent “loose” as shown in Fig. 2. These results are considered as an indication of the raised awareness among people about the importance of proper application of the mask on the face, not to be loose, but at the same time comfortable as the highest percentage of the study subjects tend to use surgical masks and to wear without leaving a gap and without a frequent need to adjust it for the time it is worn on the face so it can be worn without slipping and it does not require to be touched frequently.

Although it is known that surgical and cloth masks are characterized by being loose-fitting and N95 masks are characterized by being tight-fitting [13]. The study subjects described the adaptation (fit) of the mask on their face according to their own self-perception in applying it on their face, not in terms of air leakage from the edges of the mask.

The highest percentage of the study subjects has high voice demand per working hours. Only (2.6%) of the study subjects have less than 25% voice demand per working hours as shown in Table 2. For more clarification of the nature of the subjects’ speech usage during work, the “Levels of Speech Usage Categorical Rating Scale” was used and it was described by (58.8%) of the study subjects as being “routine,” while in 34.6% of the study subjects, it was described as being “extensive,” and in the minority of the study subjects, it was described as being “intermittent,” as shown in Table 3. The results of voice demand and speech usage during work indicate that most of the subjects under the study have moderate to high voice demand during work and the highest percentage of the study subjects use their speech in routine and extensive ways.

It was important to evaluate the subjects’ working environment and the workplace condition to have an idea about the factors present in the workplace that might contribute to increase or decrease the level of voice demand and speech usage, which include general factors such as the health condition of people the subjects deal with, presence of sources of noise, and presence of acoustic measures to reduce the noise in the workplace and specific factors added during the pandemic period as measures to reduce the risk of infection, such as the presence of transparent screening panels or keeping a safe social distance.

Regarding the main health condition of people the subjects deal with, it was found that half of the study subjects deal mainly with individuals suffering from communication disorders, or other medical conditions including neurological, psychiatric, and ophthalmological disorders as shown in Fig. 3. This points to that nearly half of subjects under the study deal with people having disorders that might require the subject to exert more effort during communication.

Regarding the frequency of exposure to noise in the workplace, Almost all the subjects reported the presence of noise during work with different frequencies, apart from (2%) who did not report the presence of noise during work as shown in Table 4. Workplace noise is considered as one of the most important factors negatively affecting effective communication. Mendel et al. [14] stated that regardless of whether a surgical mask is present or not, noise had a negative effect on speech understanding; especially in listeners suffering from hearing impairment.

Regarding the acoustic treatment methods to reduce the noise in the workplace, only (29.5%) reported the presence of measures to reduce the noise in the workplace, including sound absorption and to lesser extent partitions between desks as shown in Fig. 4. These results indicate that the working environment of most of the subjects under the study has a poor acoustic condition with no measures to reduce noise which considered one of the factors that might have a negative impact on the subjects’ communication in such working environments.

Regarding the workplace modifications during the pandemic, 47.7% of the subjects reported the application of a safe social distance as a strict measure in the workplace, and 3.9% reported using personal protective equipment which is considered as a precaution taken by the study subjects, not a modification to the workplace as shown in Fig. 5. Although the social distance is an important measure to control the spread of infection and one of the United States Centers for Disease Control and Prevention guidelines, it is expected to negatively affect communication in the workplace. However, it should be mentioned that in some workplaces such as emergency rooms and operation rooms and it was difficult for the subjects to keep a safe social distance during work.

The significant increase in self-perception of voice fatigue in case of wearing face masks as shown in Table 5 can be attributed to that the speaker usually tends to use compensatory mechanisms and vocal adjustments to make the speech more clear and understandable for the listeners. This is in agreement with the findings of Ribeiro et al. [1] who classified their participants into two groups: the working group who wore face masks for professional and essential activities during the pandemic; and the essential activities group, who wore face masks only for essential activities during the pandemic. They used the Vocal Fatigue Index (VFI) to evaluate the perception of vocal fatigue while wearing the face mask and found significantly higher scores of vocal fatigue symptoms in the working group than the essential activities group. The same group showed higher frequency and intensity of vocal tract discomfort while wearing face masks when the Vocal Tract Discomfort Scale was applied.

Regarding the voice measures, shimmer in dB and mean fundamental frequency (MFF) were significantly increased when measured with a face mask than without a face mask. Noise to the harmonic ratio (N/H ratio) and maximum phonation time (MPT) were significantly decreased when measured with a face mask than without a face mask. Further analysis based on gender distribution revealed that the effect of wearing face masks on males was evident in the increase of the value of N/H ratio, and the effect on females was evident in the increase in value of shimmer and in the decrease of MPT with the use of face masks as shown in Tables 6, 7, and 8. The increase in the MFF with a face mask may be because the study subjects have perceived their speech as less intelligible when wearing face masks, driving them to increase their pitch and loudness to make their voices sound clearer. In addition, a previous study has found that the fundamental frequency can be affected by different factors including the subject’s age, length of vocal folds, and language [15]. Brockmann et al. [16] stated that vowels, gender, voice sound pressure level, and fundamental frequency, each had significant effects either on jitter or on shimmer, or both. The decrease in the MPT with a face mask can be explained by the difficulty to control breathing associated with wearing a face mask, as previously shown in the section on self-perception of breathing difficulty in the current study.

The subtest scores and the total score of the ASIT-AA were significantly decreased with a face mask than without a face mask as shown in Table 9. These results indicate that face masks have a negative impact on the speech intelligibility even in absence of background noise, as the face masks act as barriers that cause speech quality degradation. In addition, they cover the lower part of the face, so they interfere with the visual cues. This makes the understanding of speech a more challenging task.

The score of the speech intelligibility test was significantly decreased regarding the n95 mask than a surgical mask. Also, the score was significantly decreased regarding the n95 mask than the cloth mask. These results indicate that the n95 mask has a more adverse effect on speech intelligibility than the surgical mask and cloth mask. The results of the current study are consistent with the results of Goldin et al. [17] who stated that face masks cause acoustic degradations by acting as low-pass filters that attenuate high frequencies (between 2000 and 7000 Hz) by about 3 to 4 dB (dB) for surgical masks and up to 9 to 12 dB for N95 masks.

However, the results of the current study are contrary to the results of a previous study done by Mendel et al. [14] who assessed the effect of the surgical mask on speech understanding using the Connected Speech Test (CST). They showed different results than the current study as they found that the surgical mask did not have a significant effect on speech understanding. The difference between their results of and the results of the current study could be explained by the fact that words in sentences can be comprehended more accurately than the same words in isolation, as masked sounds may be expected from the wider language context.

No significant difference between the three types of face masks regarding the scores of self-perception of voice fatigue, self-perception of speech unintelligibility with a face mask, and the received auditory feedback with a face mask as shown in Table 10. These results indicate that subjects’ self-impression about speech intelligibility is the same regardless of the type of the mask used by the subjects. They used compensatory strategies in order to produce a more intelligible speech regardless of the type of mask they used. The auditory feedback can be affected by additional multiple factors such as the presence of background noise in addition to the listener’s age, hearing abilities, and other health problems.

Only the MFF, was significantly increased in case of using an n95 mask than in case of using a surgical mask, and also, it was significantly increased in case of using the n95 mask than in case of using a cloth mask as shown in Table 11. Further analysis according to gender declared that this change was evident in male participants. Results of the current study are in partial agreement with the findings of Cavallaro et al. [18] and Fiorella et al. [19], who had similar results to the current study regarding the measures of jitter; as they found no significant difference in jitter between the two different conditions (with a surgical mask and without surgical mask). However, their results differed from the current study regarding the measures of the fundamental frequency, shimmer, and harmonics-to-noise ratio (HNR); as they found no significant difference between the two different conditions (with a surgical mask and without a surgical mask). The results of the current study are also in partial agreement with Lin et al. [20], with similar results regarding the measures of the fundamental frequency. They found increasing trends without statistical significance in the condition with surgical masks relative to without a surgical mask. However, their results differed from the current study regarding the measures of jitter, shimmer, N/H ratio, and MPT as they found that while wearing medical masks, both jitter and shimmer significantly decreased, the MPT showed increasing trends without statistical significance, and the N/H ratio showed no significant change. They stated that the improvements in jitter and shimmer may result from the increases in fundamental frequency and intensity. In addition, the effect of medical masks as a filter may to some extent play a role in the decrease of jitter and shimmer.

The current study included a larger number of participants than the previous ones with different mean ages of the subjects under the study. The current study also assessed voice parameters using not only the surgical mask but also the cloth mask and the N95 mask. It is possible that the subjects unconsciously used an individual strategy to adapt their phonation style, which may account for the findings. Although the subjects were instructed to keep their phonation style constant at their comfort level, it was difficult to monitor this. Adaptation while wearing a face mask may include an unconsciously change in vocal projection to compensate for the presence of the mask.

The score of self-perception of speech unintelligibility and the score of the received auditory feedback with a face mask were significantly increased in medical professions than in non-medical professions as shown in Table 12. It should be mentioned that most of the subjects working in medical professions deal mainly with people having either communication disorders or medical disorders that may affect communication. Dealing with people with such conditions, especially in the presence of adverse environmental conditions as seen in the findings of the current study, requires the subject to intentionally use more compensatory mechanisms as a response to the auditory feedback from the listeners or as the subject feels that he needs to use such mechanisms in order to compensate for the presence of these obstacles (wearing a face mask and adverse environmental conditions) to achieve effective communication. Furthermore, medical staff needs to use more clear voice when dealing with patients in order to convey information about their medical condition clearly, to get their attention, show empathy and reassure them.

The results shown in Table 13 indicate that the questionnaire showed content consistency according to Cronbch’s alpha score. There is also a high correlation between the test–retest results thus indicating a high reliability of the questionnaire.

## Conclusion

The study revealed that wearing face masks, regardless of the mask type, has a negative impact on communication in the workplace, especially with poor room acoustics, as they affect speech intelligibility on both subjective and objective aspects, with subsequent affection of voice as it was found that they caused increased self-perception of voice fatigue and changes in some aspects of objective voice parameters. The study also highlighted that the use of N95 masks may have more adverse effects on speech intelligibility than the surgical and cloth masks, in addition to the difficulty faced by individuals working in medical professions during communication with their patients as regards self-perception of speech unintelligibility and the received auditory feedback with face masks.

### Recommendations

From the results of the current study, it is recommended to modify the workplace targeting improvement of the acoustic condition as it will enhance effective communication by reducing the self-perception of speech unintelligibility, limiting the need to use compensatory mechanisms, and consequently, the self-perception of vocal fatigue symptoms will decrease. Occupational voice users should be encouraged to follow voice hygienic measures to overcome the effect of voice fatigue resulting from the compensatory mechanisms used while wearing the face mask during work. The use of N95 masks is not preferred in professions with high vocal demand, provided that other protective measures are available in the workplace.

## Data Availability

The datasets used or analyzed during the current study are available from the corresponding author on reasonable request.

## Appendix 1

### History taking to collect personal data and data about the workplace.

**Table:**

➣Personal data:	
Age: (years)	
Gender:	1. Male
	2. Female
Educational level:	1. Diploma
	2. University education
	3. Postgraduate studies
Profession:	1. Medical profession
	2. Non-medical profession
Daily workload: (hours/day)	
Weekly workload: (days/week)	
Face mask type:	1. Surgical
	2. Cotton/Cloth
	3. N95
Adaptation of the mask on the face:	1. Loose
	2. Comfortable
	3. Tight
Voice demand during work: (percentage of voice use per working hours)	1. 0–25%
	2. 25–50%
	3. 50–75%
	4. 75–100%
Speech usage during work:	1. Undemanding
	2. Intermittent
	3. Routine
	4. Extensive
	5. Extraordinary

**Table:**

➣Workplace:	
Number of people in the office:	1. 2–5
	2. 6–20
	3. 21–100
	4. > 100
The main age-group you deal with: (years)	1. 0–12
	2. 13–19
	3. 20–64
	1. ≥ 65
The main type of people you deal with:	1. Normal/healthy
	2. Communication disorders
	3. Other disorders
Workplace noise:	0. Never
	1. Sometimes
	2. Usually
	3. Always
Source/s of noise:	1. People chatting
	2. Machines
	3. Traffic
	4. Other
	5. None
	6. More than one answer
Presence of acoustic treatment method/s to reduce noise:	1. Sound absorption on walls
	2. Partitions between desks
	3. Carpets
	4. Other
	5. None
	6. More than one answer
Workplace modification/s after the pandemic:	1. Transparent screening panels
	2. Social distance
	3. Other
	4. None
	5. More than one answer

## Appendix 2

### Self-perception of voice fatigue

**Table:**

➣ Voice fatigue without face mask:	
My voice gets hoarse with voice use:	0. Never
	1. Sometimes
	2. Usually
	3. Always
I tend to generally limit my talking after a period of voice use:	0. Never
	1. Sometimes
	2. Usually
	3. Always
It is effortful to produce my voice after a period of voice use:	0. Never
	1. Sometimes
	2. Usually
	3. Always
My voice feels weak after a period of voice use:	0. Never
	1. Sometimes
	2. Usually
	3. Always
I experience throat pain at the end of the day with voice use:	0. Never
	1. Sometimes
	2. Usually
	3. Always
I experience discomfort in my neck with voice use:	0. Never
	1. Sometimes
	2. Usually
	3. Always
My voice feels better after I have rested:	3. Never
	2. Sometimes
	1. Usually
	0. Always

**Table:**

➣ Voice fatigue with face mask:	
My voice gets hoarse with voice use:	0. Never
	1. Sometimes
	2. Usually
	3. Always
I tend to generally limit my talking after a period of voice use:	0. Never
	1. Sometimes
	2. Usually
	3. Always
It is effortful to produce my voice after a period of voice use:	0. Never
	1. Sometimes
	2. Usually
	3. Always
My voice feels weak after a period of voice use:	0. Never
	1. Sometimes
	2. Usually
	3. Always
I experience throat pain at the end of the day with voice use:	0. Never
	1. Sometimes
	2. Usually
	3. Always
I experience discomfort in my neck with voice use:	0. Never
	1. Sometimes
	2. Usually
	3. Always
My voice feels better after I have rested:	3. Never
	2. Sometimes
	0. Usually
	0. Always

**Table:**

II -Self-perception of speech unintelligibility:	
➣ With face mask, when I talk to people:	
• I feel that:	
Articulatory movement is difficult or restricted:	0. Never
	1. Sometimes
	2. Usually
	3. Always
My speech is unintelligible:	0. Never
	1. Sometimes
	2. Usually
	3. Always
• I try to:	
Raise my voice:	0. Never
	1. Sometimes
	2. Usually
	3. Always
Clarify my speech: (exaggerate articulators movement and/or use short phrases)	0. Never
	1. Sometimes
	2. Usually
	3. Always
Repeat what I’ve said:	0. Never
	1. Sometimes
	2. Usually
	3. Always
Slow my speech rate:	0. Never
	Sometimes
	2. Usually
	3. Always

**Table:**

III-The received auditory feedback:	
➣ With face mask, when I talk to people:	
• They comment that:	
My speech is unintelligible:	0. Never
	1. Sometimes
	2. Usually
	3. Always
• They ask me to:	
Raise my voice:	0. Never
	1. Sometimes
	2. Usually
	3. Always
Clarify my speech:	0. Never
	1. Sometimes
	2. Usually
	3. Always
Repeat what I’ve said:	0. Never
	1. Sometimes
	2. Usually
	3. Always
Slow my speech rate:	0. Never
	1. Sometimes
	2. Usually
	3. Always

**Table:**

IV-Breathing with face mask:	
Difficult inspiration:	0. Never
	1. Sometimes
	2. Usually
	3. Always
Difficult expiration:	0. Never
	1. Sometimes
	2. Usually
	3. Always

## Abbreviations

SARS-COV 2: The severe acute respiratory syndrome coronavirus 2
MFF: Mean Fundamental Frequency
MPT: Maximum phonation time
ASIT-AA: Arabic Speech Intelligibility Test for Adolescents and Adults
